# Younger adults tolerate more relational risks in everyday life as revealed by the general risk-taking questionnaire

**DOI:** 10.1038/s41598-022-16438-2

**Published:** 2022-07-16

**Authors:** Wai Him Crystal Law, Shinya Yoshino, Chun Yuen Fong, Shinsuke Koike

**Affiliations:** 1 Center for Evolutionary Cognitive Sciences, Graduate School of Art and Sciences, The University of Tokyo, Meguro-ku, Tokyo, 153-8902 Japan; 2 Faculty of Letters, Arts and Sciences, Waseda University, Shinjuku-ku, Tokyo, 162-8644 Japan; 3 International College of Liberal Arts (iCLA), Yamanashi Gakuin University, 2-7-17 Sakaori, Kofu, Yamanashi 400-0805 Japan; 4 University of Tokyo Institute for Diversity and Adaptation of Human Mind (UTIDAHM), Meguro-ku, Tokyo, 153-8902 Japan; 5 University of Tokyo Center for Integrative Science of Human Behavior (CiSHuB), 3-8-1 Komaba, Meguro-ku, Tokyo, 153-8902 Japan; 6 The International Research Center for Neurointelligence (WPI-IRCN), Institutes for Advanced Study (UTIAS), University of Tokyo, 7-3-1 Hongo, Bunkyo-ku, Tokyo, 113-8654 Japan

**Keywords:** Psychology, Risk factors

## Abstract

A range of self-report questionnaires were developed to quantify one’s risk-taking (RT) tendency. Exploring people’s perceived risk level associated with negative risk behaviors is essential to develop a better understanding and intervention policies for RT. In the present study, we proposed a 2 × 10-item scale, namely, the general risk-taking questionnaire (GRTQ), to evaluate RT tendency and risk attitude among the general population by measuring people’s engagement in and perceptions toward 10 commonly known risky behaviors. A total of 2984 adults residing in 10 prefectures in Japan (age range = 20–59, 53.12% female) provided valid responses to an online survey. Apart from the factor analysis procedures, multivariate negative binomial regression models have been applied to investigate the relationship between RT engagement and perception. We obtained two identical factors, namely, personal risk and relational risk, for both scales of the GRTQ. Increased levels of RT engagement were found in younger, male, nonmarried, nonparent and urban respondents. Despite an overall negative correlation between RT engagement and perception, our model revealed a weaker linkage in the younger population for relational risk behaviors. Overall, we showed evidence that the GRTQ is an easy-to-administer, valid and reliable measure of RT for future clinical research.

## Introduction

Behaviors that are known to have possible adverse effects on actors’ health and social and financial status are termed risk behaviors (RBs); examples of conventional RBs include illicit drug use and unsafe sexual behavior. If a person acknowledges the potential negative consequences of an act, despite being uncertain about how likely those negative outcomes would happen, still decides to engage in it, he/she is said to practice (conscious) risk-taking (RT)^1–4^. What causes someone to be involved in RT is an interesting research question. However, before investigating the underlying factors, it is necessary for researchers to accurately index the tendency/frequency of someone to commit RT^5–11^.

A behavior may be perceived as an RB by one individual but not the other since everyone would have their own subjective judgment on the probabilities of potential consequences and outcomes associated with an act, which is also known as risk perception^12^. Traditionally, researchers include behavior items that they themselves perceived as RBs in questionnaires, which were then used to assess RT tendency by measuring one’s frequency of engaging in them. These scales might not be truly indexing RT since they were based on a bold assumption that participants and researchers shared the same risk perception^1,13–16^. For instance, if participants engaged in a conventional (researcher perceived) RB, such as smoking, because (1) they did not perceive it to be associated with any potential negative consequences at all, then the frequencies of smoking reported would simply reflect the prevalence of a behavior rather than the true (conscious) RT. Apart from this, one’s engagement in it could be that the person (2) believes the undesirable consequence is unlikely to happen, i.e., low level of risk perceived, or (3) does perceive an act as risky but prefers the risk, i.e., a risk-seeking attitude. For these reasons, without an individual’s self-declared risk perception of a behavior, despite the success of finding consistent linkages between engagement in conventional RBs and certain personality traits^17^, life experiences^18^, mental health conditions^19,20^ and sociodemographic factors^1^, it has been challenging for researchers to interpret the findings.

Another recently challenged assumption is that individuals or groups were categorized as risk-avoiding or risk-seeking along a continuum based on their risk attitude (aka “risk preference”)^21,22^. Under the stable risk attitude trait assumption, if male respondents were found to have a more “risk-seeking” attitude than females, they would be expected to engage in more RT across all domains, ranging from health (e.g., alcohol abuse) to recreational (e.g., bungee jumping) or relational (e.g., breaking a promise) RT than females. Empirical findings, however, did not support this notion, with conflicting RT tendencies reported within individuals/groups across different behavioral contexts^16,23–26^. For example, an individual who is willing to take risks in the relational domain, such as breaking a promise, may not feel comfortable committing actions that put one’s health on risk (e.g., drug abuse). The observations of domain-specific RT highlight the importance of exploring the latent constructs/domains underlying engagement in different RB items.

Additionally, health and risk behavioral research tended to focus highly on the behaviors that were known to bring serious negative consequences (e.g., economic, psychological and health harms) at both the individual and societal levels^27–29^. Common examples include substance use (drug/alcohol)^30,31^, risky sexual behaviors^32^, or violence^33^. Little attention has been given to general RBs, behaviors associated with “seemingly less severe” undesirable outcomes but are commonly perceived as RBs by the general population, such as riding a bicycle with the light off at night, making a dash to train doors, or breaking a promise. These general RB items make a scale’s internal reliability less susceptible to the influence of different sample characteristics, such as age, gender, education levels and cultural backgrounds, because of the low level of specific knowledge or experience required to understand the scenario and context of the RB items. A meta-analysis revealed that the measurement accuracy of the domain-specific risk-taking (DOSPERT) scale was influenced by the different degrees of familiarity respondents had with the contents of the items/situations^34^. For instance, the Cronbach’s α of the questions in the social domain was lower (lower internal consistency) for students than for nonstudents—probably due to the unfamiliarity of the students with the workplace-related items. Some ethical and financial domains also contained items (e.g., “Taking some questionable deductions on your income tax return.”) that are not general situations/decisions that one has to made across lifespan, and respondents may lack the conceptual and/or experimental knowledge of the items^34^.

The present study seeks to investigate RT using a novel questionnaire named the general risk-taking questionnaire (GRTQ), which contains general RBs in everyday decision-making and two subscales that measure one’s risk engagement (GRTQ-E) and perception (GRTQ-P). Taking into account that people across the lifespan need to make important decisions under uncertainty and risk, a sample with a broad age range was recruited in the current study. Risk attitudes in the population could be inferred from the relationship between the frequency of engagement and the associated perceived risk. In addition, the associations between engagement in the GRTQ items and other potential explanatory variables, such as age, gender, socioeconomic and other demographic characteristics, were also explored using generalized linear models (GLMs).

## Results

### Mean score differences across demographic subgroups

Higher mean scores on the GRTQ-E were observed in males than in females (1.43 vs. 1.35, *p* < 0.001, see Table 1), younger age groups (aged 20–25: 1.50 to aged ≥ 56: 1.29, *p* < 0.001), and subjects living in predominantly urban areas than in those residing in intermediate areas (1.41 vs. 1.37, *p* = 0.001). Respondents who were married (1.36 vs. 1.43, *p* < 0.001) and with children (1.34 vs. 1.42, *p* < 0.001) reported lower GRTQ-E mean scores than their counterparts.

**Table 1:** Mean score differences in the GRTQ-E and GRTQ-P with SD in parentheses (n = 2984).

	GRTQ-engagement		GRTQ-perception	
	Mean (SD)	*p*	Mean (SD)	*p*
**Gender**		< 0.001		< 0.001
Male	1.43 (0.45)		2.82 (0.54)	
Female	1.35 (0.36)		2.91 (0.51)	
**Age group (Years)**		< 0.001		0.440
20–25	1.50 (0.52)		2.83 (0.50)	
26–31	1.46 (0.47)		2.84 (0.52)	
32–37	1.42 (0.42)		2.86 (0.54)	
38–43	1.38 (0.38)		2.85 (0.55)	
44–49	1.35 (0.36)		2.88 (0.53)	
50–55	1.30 (0.30)		2.89 (0.51)	
≥ 56	1.29 (0.31)		2.90 (0.53)	
**Marital status**		< 0.001		0.015
Not married	1.42 (0.41)		2.84 (0.53)	
Married	1.36 (0.40)		2.89 (0.52)	
**Parenthood**		< 0.001		< 0.001
Without child	1.42 (0.42)		2.83 (0.53)	
With child	1.34 (0.38)		2.91 (0.52)	
**Living area**		0.003		0.985
Predominantly urban	1.41 (0.42)		2.86 (0.54)	
Intermediate	1.37 (0.39)		2.87 (0.52)	
Predominantly Rural	1.37 (0.38)		2.87 (0.50)	
**Education (ISCED levels)**		< 0.001		0.402
1	2.07 (0.50)		2.53 (0.40)	
2	1.59 (0.65)		2.88 (0.53)	
3	1.37 (0.39)		2.86 (0.52)	
4–5	1.33 (0.34)		2.86 (0.50)	
6	1.40 (0.41)		2.87 (0.55)	
7–8	1.52 (0.53)		2.85 (0.54)	
**Household Income (million yen/year)**		0.131		0.917
< 4	1.40 (0.40)		2.86 (0.53)	
4 to < 8	1.37 (0.41)		2.87 (0.53)	
8 to < 12	1.39 (0.39)		2.87 (0.53)	
12 or above	1.42 (0.50)		2.88 (0.51)	

Group differences were compared by Mann–Whitney U-test, Kruskal–Wallis test and Dunn Test for variables (tied ranks adjusted). All tests were two-tailed, with an alpha level of .05. All group differences remained significant after Bonferroni-adjustment for multiple testing except marital status in GRTQ-P. There were no missing data for all variables.

For the GRTQ-P, females had higher mean scores than males (2.91 vs. 2.82, *p* < 0.001), and respondents with children associated greater risk to GRTQ items than those who were childless (2.91 vs. 2.83, *p* < 0.001).

### Bivariate correlations between GRTQ-E and GRTQ-P

The correlation between risk engagement and perception could represent a sample’s risk attitude, potentially highlighting how they relate risk with their corresponding behaviors. An identical 2-factor structure, namely, Personal risk and Relational risk, for both the GRTQ-E and GRTQ-P was revealed and confirmed by exploratory and confirmatory factor analysis (details on the development and validity of the GRTQ can be found in the Supplementary Information online). Bivariate correlation analysis revealed that the mean scores of the GRTQ-E were negatively correlated with those of the GRTQ-P for the full scale (τ_b_ = − 0.223) and the Personal Risk (τ_b_ = − 0.241) and Relational Risk (τ_b_ = − 0.183) subscales (see Table 2).

**Table 2:** Kendall's tau *b* correlations (τ_b_) between the full and subscales of the GRTQ-E and GRTQ-P derived from the factor analysis (n = 2984).

	GRTQ-E	GRTQ-P			GRTQ-E
	Full	Full	Personal risk	Relational risk	Personal risk
**GRTQ-P**					
Full	− 0.223***				
Personal risk	− 0.238***	0.736***			
Relational risk	− 0.131***	0.641***	0.314***		
**GRTQ-E**					
Personal risk	0.647***	− 0.155***	− 0.241***	0.009	
Relational risk	0.800***	− 0.215***	− 0.188***	− 0.183***	0.340***

***p < 0.001. Correlations representing Risk Attitude were in Bold.

### GLM: explanatory variables for the GRTQ-E in personal and relational RBs

#### Crude associations (controlling for age and gender effects only)

Mean frequency counts of the GRTQ-E Personal Risk and Relational Risk subscales and the incidence rate ratios (IRR: mean ratio of the outcome) of the crude association models on them are presented in Table 3. Three categorical variables were significantly associated with the frequency counts of the GRTQ-E Personal Risk subscale. Male gender (IRR = 1.578, *p* < 0.001), urban place of residence (IRR = 1.094, *p* < 0.01) and 12 million yen annual household income (IRR = 1.402,* p* < 0.01) were all linked with greater GRTQ-E Personal Risk scores. For the GRTQ-E Relational Risk subscale, male gender (IRR = 1.161, *p* < 0.001) was associated with greater GRTQ-E scores than female gender, and being a parent was found to be linked with a lower level of GRTQ-E (IRR = 0.896, *p* < 0.01).

**Table 3:** Crude Association Models: Potential variables associated with the GRTQ-E Personal Risk and Relational Risk scores among all participants (n = 2984).

	*N%*	Frequency counts of GRTQ-E: Personal Risk		Frequency counts of GRTQ-E: Relational Risk	
		Mean	IRR^a^	Mean	IRR^a^
**Age**		–	0.979***	–	0.984***
**GRTQ-P**					
Personal risk		–	0.872***	–	–
Relational risk		–	–	–	0.943***
Education (ISCED levels)		–	1.007^ns^	–	1.007^ns^
**Gender**					
Male	46.88	1.78	1.578***	2.54	1.161***
Female	53.12	1.19	1 (ref)	2.29	1 (ref)
**Married**					
Yes	50.47	1.37	1.051^ns^	2.20	0.942^ns^
No	49.53	1.56	1 (ref)	2.61	1 (ref)
**Being a parent**					
Yes	40.28	1.28	0.932^ns^	2.10	0.896**
No	59.72	1.59	1 (ref)	2.61	1 (ref)
**Living area**^**b**^					
Predominantly Rural	46.34	1.20	0.835^ns^	2.46	1.046^ns^
Intermediate	45.88	1.45	0.941^ns^	2.32	0.969^ns^
Predominantly Urban	7.77	1.62	1.094**	2.48	1.024^ns^
**Household income**^**b**^** (million yen/year)**					
< 4	42.02	1.45	0.974^ns^	2.51	1.027^ns^
4 to < 8	40.75	1.40	1.058^ns^	2.33	0.975^ns^
8 to < 12	12.57	1.53	1.058^ns^	2.32	0.989^ns^
12 or above	4.65	1.91	1.402**	2.34	1.009^ns^

*IRR* Incidence-Rate Ratio.

^a^Controlled by age and gender.

^b^Weighted effect coded because of the highly unbalanced group size.

^ns^Not significant.

**p < 0.01, ***p < 0.001, Variables with *p* < 0.01 are in bold.

Two-dimensional variables, namely, age and GRTQ-P ratings, were significantly negatively associated with the GRTQ-E in both the Personal Risk (age: IRR = 0.979; GRTQ-P Personal Risk: IRR = 0.872) and Relational Risk (age: IRR = 0.984; GRTQ-P Relational Risk: IRR = 0.943) subscales, all *p*s < 0.001. The IRRs indicated that the rate ratio for engagement in the GRTQ-E Personal Risk and Relational Risk subscales would be expected to decrease by a factor of 0.979 and 0.984 per year increase in age and decrease by a factor of 0.872 and 0.943 per score increase in the GRTQ-P, respectively.

#### Multivariate models (all independent variables mutually adjusted)

Multivariate negative binomial regressions revealed that all explanatory variables of the GRTQ-E discovered in crude association models for both subscales remained significant when all independent variables were mutually adjusted (see Table 4). Men had a greater GRTQ-E score on both subscales (Personal Risk: IRR = 1.339, Relational Risk: IRR = 1.178; both *p*s < 0.001). Living in predominantly urban areas (IRR = 1.088, *p* = 0.007) and with an annual household income of > 12 m yen (IRR = 1.355, *p* = 0.017) remained associated with a higher engagement in the GRTQ-E Personal Risk subscale. Being a parent remained linked with a lower GRTQ-E on the Relational Risk subscale (IRR = 0.916, *p* = 0.042). Age was significantly negatively associated with the GRTQ-E in both the Personal Risk and Relational Risk subscales (age: IRR = 0.983 and 0.983, respectively; both *p*s < 0.001). Notably, the GRTQ-P ratings not only remained negatively related to the GRTQ-E but also had even lower IRRs for both subscales when socioeconomic, education and demographic factors and interaction effects were controlled for (Personal Risk: IRR = 0.869, Relational Risk: IRR = 0.932; both *p*s < 0.001).

**Table 4:** Multivariate negative binomial regression model estimates (n = 2984), modeling the GRTQ-E Personal Risk and Relational Risk subscales.

	Frequency Counts of GRTQ-E: Personal Risk					Frequency Counts of GRTQ-E: Relational Risk				
	*B (SE)*	z	*p*	IRR	Δ%	*B (SE)*	z	*p*	IRR	Δ%
Age	− **0.017 (0.004)**	− **4.44**	** < 0.001**	**0.983**	− **1.71**	− **0.017 (.002)**	− **8.43**	** < 0.001**	**0.983**	− **1.73**
GRTQ-P										
Personal risk	− 0**.140 (0.013)**	− **10.71**	** < 0.001**	**0.869**	− **13.08**					
Relational risk						− **0.070 (.008)**	− **8.71**	** < 0.001**	**0.932**	− **6.78**
Education (ISCED Levels)	− 0.002 (0.021)	− 0.11	0.915	0.998	− 0.22	0.012 (0.011)	1.11	0.265	1.012	1.23
Gender (Male)	**0.292 (0.061)**	**4.79**	** < 0.001**	**1.339**	**33.91**	**0.164 (0.032)**	**5.14**	** < 0.001**	**1.178**	**17.83**
Married (Yes)	0.098 (0.081)	1.21	0.225	1.103	10.33	0.023 (0.043)	0.53	0.596	1.023	2.30
Being a parent (Yes)	− 0.126 (0.081)	− 1.56	0.118	0.881	− 11.86	− **0.087 (0.043)**	− **2.03**	**0.042**	**0.916**	− **8.35**
Living area^a^										
Predominantly Rural	− 0.163 (0.103)	− 1.58	0.115	0.850	− 15.04	0.030 (0.052)	0.57	0.567	1.030	3.05
Intermediate	− 0.057 (0.032)	− 1.82	0.069	0.944	− 5.58	− 0.029 (0.017)	− 1.75	0.081	0.971	− 2.87
Predominantly Urban	**0.084 (0.031)**	**2.69**	**0.007**	**1.088**	**8.78**	0.024 (0.017)	1.43	0.152	1.024	2.41
Household income^a^ (million yen/year)										
< 4	− 0.008 (0.037)	− 0.20	0.838	0.993	− 0.75	0.017 (0.019)	0.90	0.370	1.017	1.75
4 to < 8	− 0.048 (0.036)	− 1.33	0.182	0.953	− 4.65	− 0.020 (0.019)	− 1.06	0.290	0.980	− 1.98
8 to < 12	0.067 (0.077)	0.87	0.383	1.069	6.93	− 0.002 (0.041)	− 0.04	0.970	0.998	− 0.15
12 or above	**0.304 (0.127)**	**2.38**	**0.017**	**1.355**	**35.46**	0.023 (0.071)	0.33	0.743	1.024	2.35
Age × gender	− 0.004 (0.005)	− 0.79	0.428	0.996	− 0.43	0.003 (0.003)	1.22	0.222	1.003	0.35
Age × GRTQ-P										
Personal risk	0.001 (0.001)	0.66	0.506	1.001	0.08					
Relational risk						− **0.003 (0.001)**	− **4.52**	** < 0.001**	**0.997**	− **0.34**
Gender × GRTQ-P										
personal risk	0.008 (0.018)	0.46	0.646	1.008	0.81					
Relational risk						0.016 (0.011)	1.44	0.151	1.016	1.62
Gender × Age × GRTQ-P										
Personal risk	− 0.001 (0.002)	− 0.70	0.481	0.999	− 0.11					
Relational risk						0.001 (0.001)	0.92	0.357	1.001	0.10

*B* unstandardized regression estimates with standard errors in parentheses, *IRR* incidence-rate ratio = EXP(B). Variables with *p* < 0.05 are in bold.

Δ% Percentage change in likelihood of engagement in RBs of the GRTQ = (IRR − 1) * 100.

^a^Weighted effect coded because of the highly unbalanced group size.

**Figure 1:**
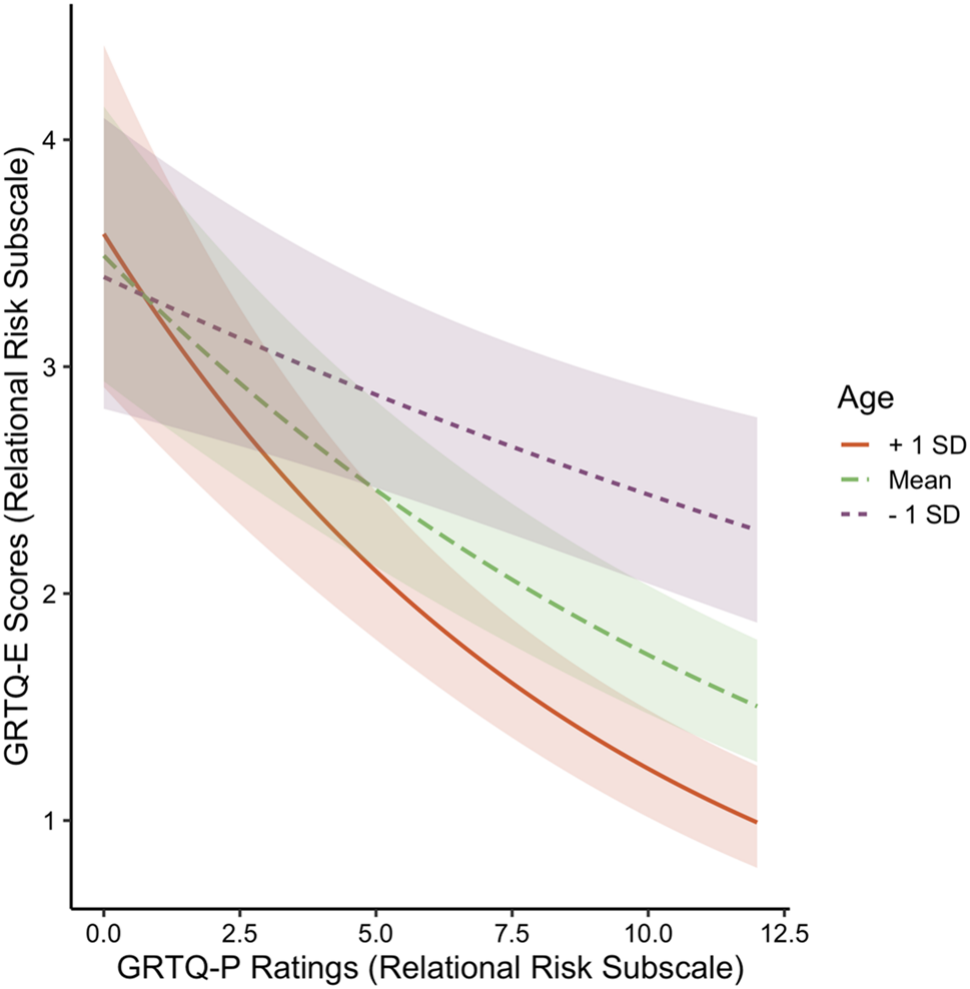
Follow-up simple slopes analysis for the interactions between age and the GRTQ-P Relational Risk ratings on the GRTQ-E Relational Risk scores. The shaded region depicts the 95% confidence level interval for the beta estimates.

Interaction effects of the three most significant explanatory variables, namely, age, gender, and the GRTQ-P ratings, were examined. Age was found to exacerbate the negative effect of the GRTQ-P on the GRTQ-E frequency for the Relational Risk subscale (IRR = 0.997, *p* < 0.001). Simple slopes analysis indicated that the negative association between the GRTQ-P and GRTQ-E for the Relational Risk subscale strengthened with aging, with a steeper slope in the older group (Fig. 1; younger (age = 29.25, mean age—1 SD): *B* = − 0.03, *z* = − 4.12; middle (40.21): *B* = − 0.06, *z* = -11.09; older (51.16, mean + 1 SD): *B* = − 0.09, *z* = − 11.05; all *p*s < 0.001).

## Discussion

The present study explored RT in a sample with a broad age range from 20 to 59 years using a novel general risk-taking questionnaire—the GRTQ. Although a few efforts to measure risk attitudes or the prevalence of risk behaviors have been made in previous studies, our study is the first to address both risk perception and actual behavioral engagement with clear evidence supporting its validity. Higher mean scores on the GRTQ-E were found in groups known to engage in more RT: male^1,35,36^, younger^27,37,38^, nonmarried^39,40^, nonparent^41^, and urban areas residing^42–45^ compared to their counterparts.

Gender and age differences were observed in both subscales of the GRTQ-E in our GLM results, with males and younger age associated with more engagement in both personal and relational RBs. These results have been frequently reported in previous literature in which risk perception was not measured, and it was concluded that males and younger respondents were more “risk-seeking” and more prone to RT^1,46,47^. However, considering the negative correlations between the GRTQ-P and GRTQ-E on both subscales, they were indeed less “risk averse”. Interestingly, the significant interaction between age and the GRTQ-P further implied that the effect of perception on engagement in the Relational Risk subscale was weaker in younger adults, confirming their stronger risk tolerance level, particularly for relational RBs. Such risk context- and gender-specific effects of age on risk preference might explain why previous RT literature, with different RBs and gender ratios in samples, reported conflicting age effects on risk tolerance (negative:^48–50^, positive:^51^).

In addition, the above findings better explained why younger participants engaged in more RT in general. First, they associated the lowest riskiness with the RB items. The negative consequences of relational RT perceived by younger adults could be less severe (less dreadful) than older adults because of the less valuable/well-established relationships they have with friends/families/colleagues, leading to a lower risk perception^52^. The differences in risk perception could also be explained by the concept of affect heuristics, which suggested that negative (positive) emotion would lead to over- (under) estimation of risk^53^. People of younger age are likely to have fewer experiences of unexpected negative consequences that induce strong negative feelings from relational RT. As a result, they might have perceived themselves to be familiar with the potential consequences associated with it and attached fewer negative feelings to relational RT, leading to an underestimation of risk for RBs in this context. The opposite idea could be applied to older people and have resulted in negative emotions associated with relational RBs, which led to an overestimation of their risk. This might also serve as a potential explanation for the effect of age on relational risk attitudes.

Second, younger adults had the lowest tendencies to avoid both personal and relational risks, which could originate from the differences in self-efficacy. Previous research has shown that people who are very competent in decision-making tend to see more opportunities, rather than threats, in a risky choice^54^. Younger adults might have higher self-efficacy and therefore engage in more RT. Combined with the dual-process theory, which suggested that younger individuals and those with higher self-efficacy might have adopted a more heuristic, rather than analytic, decision-making approach, leading to a less “rational” behavioral outcome (more RT)^55–58^. Finally, different risk attitudes and perceptions could be driven by hormonal^59^, cognitive^60^, neurological^61^, sociocultural^62^ and genetic^63^ factors, which should not be overlooked.

The study is limited by its lack of respondents aged below 20 and above 59. In addition, as part of a nationwide online survey, the applicability of the GRTQ across cultures needs to be further examined. Due to the cross-sectional nature of our data, we could not differentiate whether the differences found across age groups were caused by aging, period, or cohort effects^50,64,65^. Further investigations applying the GRTQ in longitudinal or cohort studies would help develop a deeper understanding of the effect of age, as well as its interaction with gender, on personal and relational RT. The psychometric validity of the GRTQ could be enhanced by comparing scale scores with widely established RT-related measures, including scales such as the Zuckerman Sensation Seeking Scale^66^ and the Self-Efficacy Scale^67^, behavioral tasks such as the Balloon Analog Risk Task^9^ and Columbia Card Task^68^, genotypic data^63^ and hormonal measures such as testosterone and cortisol^59^.

An individual’s decision making in everyday risk behaviors could lead to personal and public safety issues. The present study revealed that engagement in RBs was negatively correlated with the perceived level of risk people associated with the behaviors and that such linkage was weaker in the younger population (“less risk averse”). Strategies for the prevention and intervention of RBs may include education programs that emphasize their negative outcomes in the general population, with an extra focus on the objective likelihood of adverse consequences to reduce the associated safety issues among the young populations. In addition, we demonstrate the utility of a novel RT measure, the GRTQ, through its stable factor structure and measurement invariance across gender and age groups, making it a useful tool to investigate the aging, period or cohort effects when applied to longitudinal studies and the associations between RT and neurobiological, sociocultural, cognitive, or physiological measures among patients with psychiatric disorders and the general population.

## Methods

### Participants

The current study is part of an anonymous and cross-sectional online study about risk engagement and perception. A total of 3417 participants were randomly sampled from the registrants of iBRIDGE, a Japanese survey research company, between September and November 2020, stratified by geographical location (10 Japanese prefectures), age range and gender. This study was approved by the Ethics Committee of the University of Tokyo (No. 20-172). All procedures performed in this study were in accordance with the 1964 Helsinki declaration and its later amendments or comparable ethical standards. Online informed consent was obtained from all participants.

The analyses included 2984 adults who provided valid responses to the questionnaires. The mean age was 40.21 years (SD = 10.95), with 53.12% female, 50.47% married and 40.28% having child(ren) (see Supplementary Table S1 online). For the geographic locations, prefectures were categorized into predominantly urban, intermediate and predominantly rural areas based on the regional typology established by the Organisation for Economic Co-operation and Development^69^, with 46.35% and 45.88% of participants residing in predominantly urban and intermediate areas, respectively. Based on the contents of the self-indicated educational categories, education attainment was coded according to the International Standard Classification of Education (ISCED)^70^, resulting in a mean ISCED level of 4.57 (SD = 1.46), which was comparable to the general population in Japan stratified by age groups (*p* > 0.05)^71^.

Of these participants, 100 randomly selected respondents were readministered the online survey one month later to test the reliability of the scale, of which 99 provided valid responses.

### The general risk-taking questionnaire (GRTQ)

The sources of the initial 19 items for the GRTQ are listed in Table 5. RB items that are widely experienced or familiar across broad sample characteristics were selected from the YRBS (Japanese Version, 2011)^72^ and the Risk-taking Behavior Scale for Undergraduates (RIBS-U)^73^. To examine the dimensionality of RT, the selected items cover a range of previously discovered RB domains, including social, health, traffic, and financial items. With the aim of choosing items that would be likely to be perceived as RBs by our respondents, the majority of our items were taken from the RIBS-U considering that all items, retained or not in the final scale, were nominated by Japanese undergraduates as RBs at the early development stage of the RIBS-U. The final RIBS-U is a 12-item Japanese RT engagement scale that contains two subscales (factors discovered), namely, personal and social risk, with good construct validity and test–retest reliability. Another three items, which are more general health RBs, were selected from the YRBS considered the benefits of including them on an RT scale, as discussed above, and the unchanged prevalence of them over the ten years (from 2001 to 2011) despite a general trend of decrease among most of the other YRBS items.

**Table 5:** Source of the initial 19 items for the GRTQ.

Items	Source
1. Binge Drinking	RIBS-U-Personal Risk Subscale
2. Smoking (Tobacco Use)	
3. Take a shot in at a social function (Alcohol)	
4. Drive after drinking	
5. Gambling (such as Slot Machine and Horse Racing)	
6. Ignore traffic signals	RIBS-U-Social Risk Subscale
7. Lying	
8. Being late for school or meetings	
9. Play Truant	
10. Make a dash for Train doors/Rush to board a departing train	
11. Break a Promise	
12. Cheating on tests/exams	Items not retained in the final RIBS-U
13. Shoplifting	
14. Steal money or property from others	
15. Illegal Drug Use	
16. Ride a bicycle with the light off at night	
17. Take diet pills, powders, or liquids	JYRBS2011
18. Do not eat for ≥ 24 Hours	
19. Vomit or take laxatives	

All questions were distributed in pseudorandom order in the data collection process. RIBS-U: Risk-taking Behavior Scale for Undergraduates. JYRBS2011: Japanese Youth Risk Behavior Survey.

The GRTQ contains two types of questions for all items, Engagement (-E) and Perception (-P). Following the RIBS-U scale, the GRTQ-E scale asked participants, “How frequent do you normally engage in the following behaviors? Please indicate the most appropriate option for each of the behavioral items.” 4-Likert response options include “Never”, “Seldom”, “Some of the time” and “Most of the time” (Ranged 1–4, greater score indicates more frequent engagement of each risk item). The GRTQ-P scale began with the question "To what extent do you think that the following behaviors are risky considered the possible adverse effect on the actor's health, social and financial status, etc.? “. Response options are “Not at all risky”, “Slightly risky”, “Moderately risky”, and “Very risky”, again, scored as 1, 2, 3, and 4, respectively.

### Statistical analysis

#### Generalized linear models to explore variables associated with GRTQ-E

Based on the obtained dimensionality, we estimated two separate generalized linear models (GLMs) for each outcome of interest (frequency counts of the GRTQ-E for items loaded onto each factor). We first examined crude associations between the independent (GRTQ-P ratings, socioeconomic and demographic variables) and outcome variables (frequency counts of the GRTQ-E for the extracted factors) while adjusting only for gender and age effects. Then, final multivariate models, which allow all potential explanatory variables to mutually adjust, were performed. To estimate the potential moderating effect of age and gender on the GRTQ-P’s impact on GRTQ-E frequency, we added the interaction terms of age × GRTQ-P, gender × GRTQ-P, and age × gender × GRTQ-P to the final multiple regression models.

Relationships between the GRTQ-E and independent variables are indicated by the incidence rate ratios (IRR: mean ratio of the outcome) from negative binomial regressions^74^. Negative binomial regressions were chosen because of the overdispersion (i.e., model variance exceeds the mean) observed in the response variables, which were nonnegative integer responses that approximated a Poisson distribution^75–79^. IRR and unstandardized regression coefficients of the GLM models were estimated via the “mfx” (v1.2-2)^80^ and “MASS” (v 7.3-53)^81^ packages, respectively, implemented in R. The *p-*values reported in GLM analysis were not multiple testing corrected.

Significant interactions revealed from the GLM were tested and interpreted by simple slopes analysis (SSA), which considers the regression of the explanatory variable on the outcome measure for low (mean − 1 SD), average (the mean), and high (mean + 1 SD) levels of the moderating variable^82,83^. Consequently, the nature of the interaction effect was visualized and interpreted by plotting and comparing the slopes in terms of their significance and the values and directions of the unstandardized regression coefficients (*B*)^82^. SSA was performed using the “interactions” package (v1.1.3)^84^ implemented in R.

## Supplementary Information

Supplementary Information.

## Data Availability

The data are available after revision approval from the ethical review board at The University of Tokyo. Please ask the corresponding author if needed.
