# Supplementary material for: Younger adults tolerate more relational risks in everyday life as revealed by the general risk-taking questionnaire
Source: Sci Rep. 2022 Jul 16;12:12184. doi: 10.1038/s41598-022-16438-2 (PMC9288464; doi:10.1038/s41598-022-16438-2)
Supplement: Supplementary file 1 — Supplementary Information. [file 41598_2022_16438_MOESM1_ESM.pdf]

Supplementary Information

Younger adults tolerate more relational risks in everyday life as revealed by the General Risk-taking Questionnaire

Wai Him Crystal Law[1], **Shinya Yoshino**[1,2], **Chun Yuen Fong**[1,3], **and Shinsuke Koike**[1, 4-6,*]

[1] Center for Evolutionary Cognitive Sciences, Graduate School of Art and Sciences, The University of Tokyo, Meguro-ku, Tokyo 153-8902, Japan

[2] Graduate School of Letters, Arts and Sciences, Waseda University, Tokyo, 162-8644, Japan

[3] International College of Liberal Arts (iCLA), Yamanashi Gakuin University, 2-7-17 Sakaori, Kofu, Yamanashi 400-0805, Japan

[4] University of Tokyo Institute for Diversity & Adaptation of Human Mind (UTIDAHM), Meguro-ku, Tokyo 153-8902, Japan

[5] University of Tokyo Center for Integrative Science of Human Behavior (CiSHuB), 3-8-1 Komaba, Meguro-ku, Tokyo 153-8902, Japan

[6] The International Research Center for Neurointelligence (WPI-IRCN), Institutes for Advanced Study (UTIAS), University of Tokyo, 7-3-1 Hongo, Bunkyo-ku, Tokyo 113-8654, Japan

*skoike-tky@umin.ac.jp

TABLE OF CONTENT

SUPPLEMENTARY METHOD 3

SUPPLEMENTARY TABLE S1. FULL-SAMPLE AND THE SPLIT GROUP SUBSAMPLE CHARACTERISTICS. 4

Exploratory factor analysis and item reduction 6
Confirmatory factor analyses 7
Concurrent validity 7
Test-retest reliability 7

SUPPLEMENTARY RESULTS 9

Preliminary EFA of the 19-item GRTQ-E 9

SUPPLEMENTARY TABLE S2. PRELIMINARY RESULTS OF MVN, KMO, AND BARTLETT'S TESTS. 10

SUPPLEMENTARY FIGURE S1. THE SCREE PLOT OF PARALLEL ANALYSIS ON THE 15-ITEM GRTQ-E. 10

Main EFA of the 10-item GRTQ 11

SUPPLEMENTARY TABLE S3: DESCRIPTIVE STATISTICS OF THE GRTQ-E AND GRTQ-P (N = 2984). 11

Factor extraction 11

SUPPLEMENTARY FIGURE S2. THE SCREE PLOT OF PARALLEL ANALYSIS ON THE 10-ITEM GRTQ-E. 12

SUPPLEMENTARY FIGURE S3. THE SCREE PLOT OF PARALLEL ANALYSIS ON THE 10-ITEM GRTQ-P. 12

Final model – 2-Factor oblique model (10 items) and internal consistency 13

SUPPLEMENTARY TABLE S4. THE 2-FACTOR OBLIQUE PATTERN MATRIX FOR THE FINAL GRTQ-E AND -P, WITH LOADINGS OF THE WINNING MODEL OBTAINED FROM CFA LISTED. 14

CFA model fit index 15

SUPPLEMENTARY TABLE S5. CFA MODEL FIT INDEX BASED ON THE SPLIT SAMPLE FOR CFA. 17

Factor and construct validity (MI across gender and age for GRTQ-E and -P) 17

SUPPLEMENTARY FIGURE S4. RATING DISTRIBUTIONS FOR GRTQ-E AND -P ACROSS FOUR SUBGROUPS. 19

SUPPLEMENTARY TABLE S6. FIT STATISTICS OF MULTIGROUP CONFIRMATORY FACTOR ANALYSES (MG-CFA) USING THE 2-FACTOR OBLIQUE MODEL. 20

Concurrent validity 21

SUPPLEMENTARY TABLE S7. CORRELATIONS BETWEEN MEAN SCORES OF THE GRTQ-E AND RIBS-U. 21

Test-retest reliability 22

SUPPLEMENTARY TABLE S8. ONE-MONTH TEST-RETEST RELIABILITY 22

SUPPLEMENTARY REFERENCE 23

Supplementary Method

The full sample was randomly split into two subsamples: half for Exploratory factor analysis, the other half for further Confirmatory factor analysis. Independent t-tests and chi-square tests revealed no significant differences in demographic characteristic between the two split subsamples, all $p > .20$ (see Supplementary Table S1).

Supplementary Table S1. Full-sample and the split group subsample characteristics.

Sub-sample in %		Full Sample (N = 2984)	EFA Sample (n = 1492)	CFA Sample (n = 1492)		
**All**		100	50	50	χ^2/ t	*p*
**Gender**					0	1
	Male	46.88	46.85	46.92		
	Female	53.12	53.15	53.08		
**Age group (Years)**					1.20	0.230
	20-25	10.69	11.13	10.25		
	26-31	16.29	15.15	17.43		
	32-37	15.45	14.75	16.15		
	38-43	14.48	14.75	14.21		
	44-49	17.66	17.83	17.49		
	50-55	16.59	17.02	16.15		
	≥ 56	8.85	9.38	8.31		
**Marital Status**					0.03	0.855
	Not Married	49.53	49.73	49.33		
	Married	50.47	50.27	50.67		
**Parenthood**					0.50	0.478
	Without Child	59.72	60.39	59.05		
	With Child	40.28	39.61	40.95		
**Living Area**					0.11	0.946
Predominantly Urban						
	Tokyo	11.49	11.39	11.60		
	Aichi	11.63	11.39	11.86		
	Fukuoka	11.86	12.53	11.19		
	Miyagi	11.36	10.92	11.80		
Intermediate						
	Okinawa	9.08	9.72	8.45		
	Shizuoka	11.80	11.39	12.20		
	Ishikawa	9.35	9.45	9.25		
	Tottori	4.29	4.36	4.22		
	Hokkaido	11.36	11.19	11.53		
Predominantly Rural						
	Iwate	7.77	7.64	7.91		
**Education - ISCED Level (Years)**					2.42	0.877
1	Primary (6)	0.34	0.20	0.47		
2	Lower secondary (9)	1.71	1.88	1.54		
3	Upper secondary, not completed (9-11)	1.91	1.88	1.94		
3	Upper secondary (12)	33.11	33.58	32.64		
4-5	Short cycle tertiary/ tertiary not completed (12-16)	24.33	24.13	24.53		
6	Bachelor level (16)	33.85	33.65	34.05		
7-8	Master /Doctoral level (≥16)	4.76	4.69	4.83		

**Household Income (million yen/year)**				12.90	0.534
< 4	42.02	42.43	41.62		
4 to < 8	40.75	39.88	41.61		
8 to < 12	12.57	13.27	11.87		
12 or above	4.65	2.62	3.62		

Sub-sample difference in age was compared by independent sample t-test on the mean age while the other variables were compared by chi-squared test with Yate's continuity correction applied.

Exploratory factor analysis and item reduction

A preliminary EFA was first implemented on the GRTQ-E to reduce the poorly loaded items. The remaining items were subjected to another (main) EFA to obtain the finalized latent structure. To simplify the analyses, EFA on the GRTQ-P was conducted afterwards using the same items retained from the final EFA of GRTQ-E.

The violation of multivariate normality (MVN) in responses was revealed by Mardia's MVN test and Royston's MVN test using the "MVN" package (v5.8) [1] implemented in R version 3.6.3. [2]. For non-normally distributed data, inter-item correlations were checked by bivariate Kendall's tau-b (τ^b) correlation. All factor analyses in this study were based on polychoric correlation matrix considered its robustness to the skewness of ordinal variables, biased factor loadings, underestimate of the strength of linkage between items, transformation and grouping errors [3–8]. To achieve a simpler and more interpretable factor structure with an expectation of correlated factors, factors were extracted using principal axis factoring (PAF) with direct oblimin rotation.

To determine the number of factors to extract, both initial visual inspection on scree plot and parallel analysis were considered. Final number of factors to retain would be decided after re-running the EFA if needed, until achieving a factor structure that contains at least 3 items with a loading ≥ .32 and a low cross loading, and all items with >.4 communalities [9–11]. For internal consistency, ordinal alpha, Cronbach's alpha coefficients and inter-item correlations were examined [12]. Alpha value > .70 was considered as high in internal consistency [13].

Confirmatory factor analyses

The model derived by EFA was further evaluated via CFAs based on the polychoric matrices via the diagonally weighted least squares (DWLS) estimator [14–18]. All CFAs were implemented in “lavaan” package (v0.6-7) [19] implemented in R.

Three criteria were used to assess how well a model fit our data [20–24]. Two of these criteria measure relative model fit: the Comparative Fit Index (CFI) [25] and the Tucker Lewis Index (TLI) [26]. For both CFI and TLI, a model with a value of ≥ .90 is considered a reasonable fit [24,27–29]. The last criterion indicated absolute model fit (a value of zero indicates perfect fit): the standardized root mean square residual (SRMR) [30–32]. Models with a SRMR value close to .09 represents a reasonable fit and ≤ .08 indicates a good fit [20,33].

The winning model determined from the EFA and CFA was tested between four subgroups, split by gender and age (median age 40 years) as younger females (aged ≤ 40, n = 868), older females (aged > 40, n = 717), younger males (aged ≤ 40, n = 641) and older males (aged > 40, n = 758), using the full sample (n = 2984). A categorical multi-group CFA (MG-CFA) was conducted to examine MI following the guidelines provided by Pendergast and colleagues [34], via the DWLS estimator on polychoric correlation matrix [35–40].

Concurrent validity

Concurrent validity was tested using Pearson’s correlations between the scores of items originated from the two subscales of the RIBS-U (see Table 5 in main text) and corresponding subscale scores of the GRTQ-E [41,42].

Test-retest reliability

One-month test-retest reliability of the GRTQ was tested using the intraclass correlation coefficients (ICC) between baseline and follow-up data (n = 99) with a single-rating, absolute

agreement, two-way mixed-effects model [43,44]. ICC values above 0.50 were considered as having “good” [45], or “moderate” reliability [43].

Supplementary Results

Preliminary EFA of the 19-item GRTQ-E

Of the original 19 items in the GRTQ, four items, all illegal activities, correlated with each other with $\tau b \geq .80$ which indicated multicollinearity, were removed, namely “Shoplifting”, “Steal money or property from others”, “Illegal drug use” and “Drive after drinking”.

The remaining 15 items had a Kaiser-Meyer-Olkin (KMO) measure of sampling adequacy number of factors of 0.93 and significant Bartlett's Test of Sphericity ($\chi 2 = 381.7$ (df = 105), $p < .001$; see Supplementary Table S2), justifying the factorability of the dataset and hence the implementation of the EFA.

Initial visual inspection on scree plot indicated that the curve started to flatten from 2 onwards and flattened before 4 factors. However, the parallel analysis suggested a 4-factor model instead (see Supplementary Fig. S1). Based on the EFA results on the 4-factor model, 2 items with a communality below 0.4 were removed (“Gambling (such as Slot Machine and Horse Racing”, and “Smoking (Tobacco Use)”). Three items with several cross-loaders were also discarded (“Cheating in tests/exams”, “Do not eat for ≥ 24 hours”, “Ignore traffic signals”). As a result, 10 items were retained for further (i.e., the main) EFA on both GRTQ-E and GRTQ-P. The results of MVN, KMO, and Bartlett’s tests were summarized in Supplementary Table S2.

Supplementary Table S2. Preliminary results of MVN, KMO, and Bartlett’s tests.

	Pre-screen	1st EFA	2nd EFA	
**no. of Items**	19	15	10	
**Scale**	Engagement	Engagement	Engagement	Perception
**MVN**	Not Normal	Not Normal	Not Normal	Not Normal
skewness	62313**	24174**	14666**	1487**
kurtosis	484**	187**	151**	31**
H	4687**	3596**	2325**	1689**
**KMO**	*NA*	0.93	0.89	0.86
**Bartlett's Test of Sphericity (χ2)**	*NA*	381.7** (df = 105)	194.8** (df = 45)	215.6** (df =45)

*KMO: Kaiser-Meyer-Olkin (KMO) measure of sampling adequacy number of factors; MVN: Mardia’s and Royston’s Multivariate Normality tests, indicated by skewness, kurtosis and Royston’s test statistic H. The significance of these stats revealed that the items did not follow multivariate normal distribution. KMO and Bartlett’s test were not performed on the 19-item scales. ** p < .001*

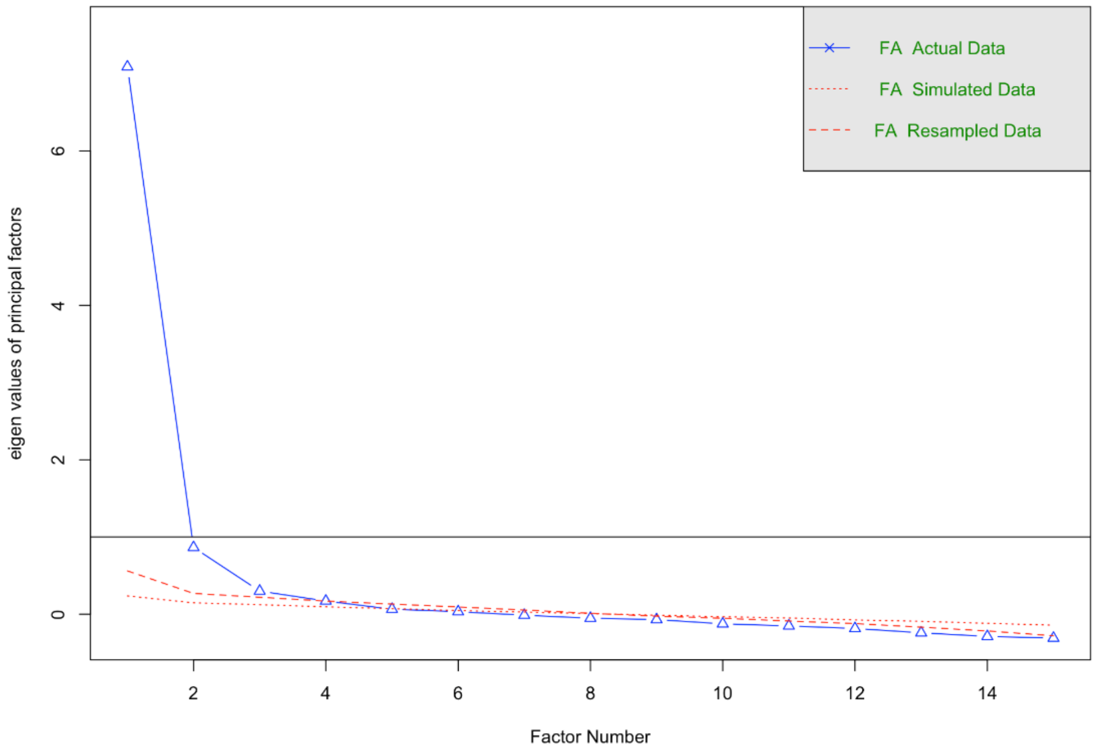

Supplementary Figure S1. The scree plot of parallel analysis on the 15-item GRTQ-E.

Main EFA of the 10-item GRTQ

Both GRTQ-E and -P mean scores were obtained by averaging the sum of ratings for the items comprising each subscale. The descriptive statistics of the 10-item GRTQ-E and GRTQ-P as well as the extracted factors were summarised in Supplementary Table S3. The overall mean score of GRTQ-E was 1.39 (SD = 0.41). With a skewness of 2.31, Kurtosis of 7.15, and a significant Shapiro-Wilk statistic, $W = 0.776$, $p < .001$, the score of GRTQ-E was considered as non-normally distributed. The mean score of GRTQ-P was 2.86 (SD = 0.53). Despite having a lower skewness (0.09) and Kurtosis (-0.23), the overall score of GRTQ-P was also non-normally distributed, $W = 0.990$, $p < .001$.

Supplementary Table S3. Descriptive statistics of the GRTQ-E and GRTQ-P (n = 2984).

	Engagement (**GRTQ-E**)			Perception (**GRTQ-P**)		
Subscale	Full	Personal Risk	Relational Risk	Full	Personal Risk	Relational Risk
N of items	10	6	4	10	6	4
Mean	1.39	1.24	1.60	2.86	3.12	2.47
SD	0.41	0.42	0.52	0.53	0.56	0.71
Median	1.30	1	1.50	2.80	3.17	2.25
Min	1	1	1	1	1	1
Max	3.90	4	4	4	4	4
Skewness	2.31	2.84	1.09	0.09	-0.37	0.38
Kurtosis	7.15	9.46	1.38	-0.23	-0.36	-0.41

Shapiro-Wilk Normality test revealed that all mean score distributions were not normal.

Factor extraction

For the GRTQ-E, scree plot and parallel analysis indicated a 2- and 4-factor model respectively (see Supplementary Fig. S2). Therefore, 4-, 3-, and 2- factor models were all explored. The 4th and 3rd factor failed to contain at least 3 items with a loading ≥.32 and hence were removed from the final model. For the same reason, the 3rd factor of GRTQ-P was removed from the final model although both parallel analysis and scree plot indicated a 3-factor model (see Supplementary Fig. S3). As a result, two factors were extracted using PAF with oblique (direct oblimin) rotation on polychoric correlation matrix.

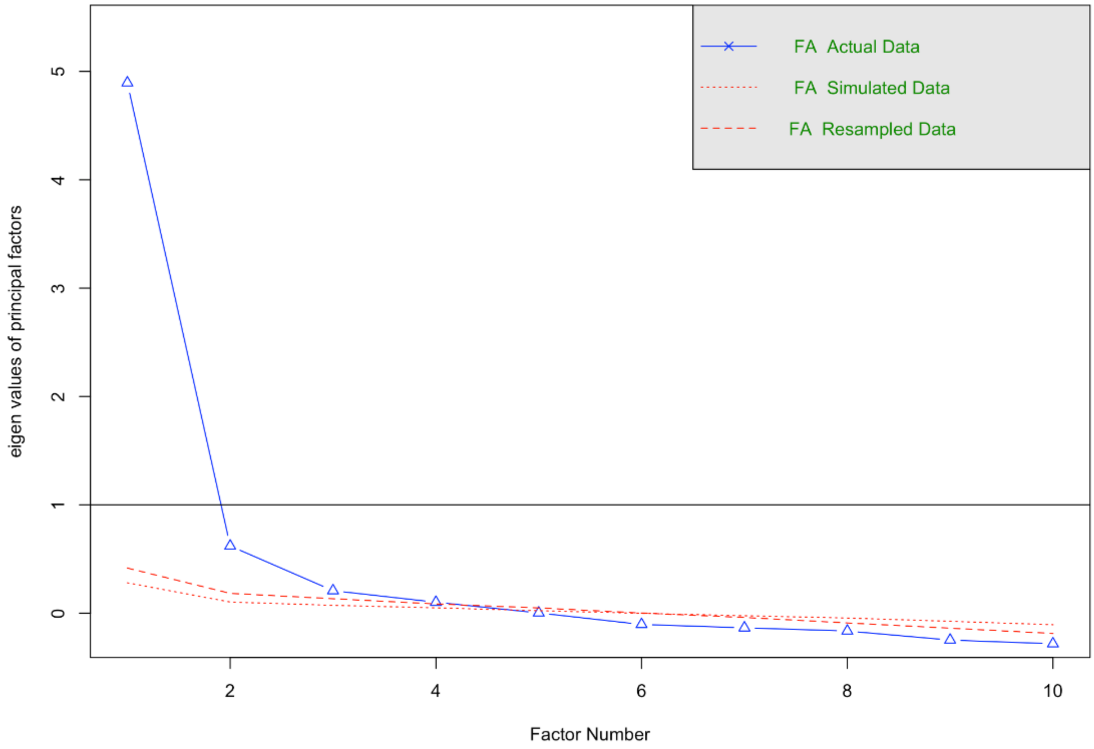

Supplementary Figure S2. The scree plot of parallel analysis on the 10-item GRTQ-E.

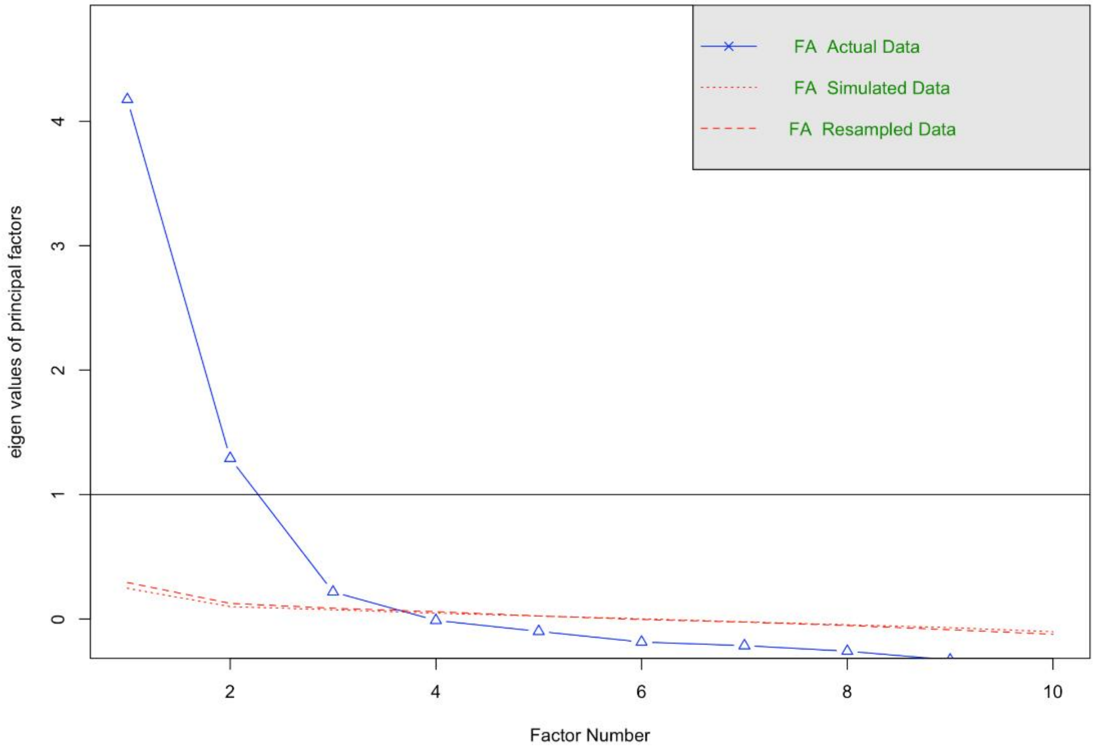

Supplementary Figure S3. The scree plot of parallel analysis on the 10-item GRTQ-P.

Final model – 2-Factor oblique model (10 items) and internal consistency

The final 2-factor model of both GRTQ -E and -P contained 10 items which all loaded saliently (≥ .32) onto one factor but not the other (see Supplementary Table S4 for the pattern matrix). None of the inter-item correlation within each scale was >.80, indicating that no items should be removed for redundancy. The models explained 58% for the GRTQ-E and 59% for the GRTQ-P of the total variance. Ordinal (and Cronbach's) alpha for the 10-item full scale of GRTQ-E and GRTQ-P were .90 (.84) and .87(.84) respectively, indicating high internal consistency for both scales. The ordinal alphas for factor 1 and 2 were also high at .89 and .83 for GRTQ-E and .84 and .90 for GRTQ-P, respectively. The use of oblique rotation was further supported by the significant correlations between the two extracted factors (GRTQ-E: .63; GRTQ-P: .38).

Supplementary Table S4. The 2-Factor Oblique Pattern Matrix for the final GRTQ-E and -P, with loadings of the winning model obtained from CFA listed.

	**GRTQ-Engagement**				**GRTQ-Perception**			
	Factor				Factor			
	1	2	Communalities	CFA loadings	1	2	Communalities	CFA loadings
**Factor: Personal Risk**								
Take a shot in drinking parties (Alcohol)	0.90		0.75	0.88		0.69	0.44	0.57
Ride a bicycle with the light off at night	0.78		0.69	0.84		0.76	0.62	0.71
Binge Drinking	0.76		0.52	0.74		0.89	0.73	0.74
Vomit or take laxatives	0.70		0.54	0.74		0.44	0.32	0.69
Take diet pills, powders, or liquids	0.69		0.50	0.69		0.41	0.35	0.66
Make a dash for Train doors/Rush to board a departing train	0.50		0.45	0.69		0.70	0.62	0.77
**Factor: Relational Risk**								
Break a Promise		0.95	0.83	0.83	0.92		0.83	0.90
Lying		0.64	0.44	0.63	0.68		0.55	0.72
Play Truant		0.56	0.58	0.79	0.91		0.78	0.84
Being late for school or meetings		0.53	0.47	0.74	0.77		0.66	0.78
Full scale Ordinal (Cronbach's) alpha	0.90 (0.84)				0.87 (0.84)			
Factor Ordinal alpha	0.89	0.83			0.84	0.90		
Factor Correlation	0.63				0.38			
Total variance explained (cumulative %)	36	58			30	59		

Note: Only Loadings with absolute value ≥ 0.32 shown.

CFA model fit index

Three models were initially examined for CFA. First, there was a one-factor model which assign all items to load onto one single factor. Second, to avoid the oversimplification of the hidden constructs, another oblique 2-factor model which allowed correlations between factors was included. Lastly, considered the previously reported low correlations among different risk domains[46], we believed that there is great need to explore the orthogonal 2-factor model which disallowed any correlations between factors. Table 4 shows the fit indices for each model. For GRTQ-E, only the 2-factor oblique model met the criteria of a reasonably good model and was considered the best model, as indicated by fit indices CFI = .956, TLI = .942 and SRMR = .049. The path coefficients (standardized factor loadings) for each item, as shown in Supplementary Table S4, were above 0.60 which further laid support to this model.

The CFA process were the same for the GRTQ-P, with the indices also shown in Supplementary Table S5. Consistent with the engagement scale, the 2-factor oblique model was the best performing model out of the three, with CFI = .941, TFI = .935 and SRMR = .083, fulfilling the general index for a reasonable model. All items loaded to the factors with

a >.50 path coefficient (standardized factor loading) for this model, as shown in Supplementary Table S4, providing additional support for the efficacy of this model.

Supplementary Table S5. CFA model fit index based on the split sample for CFA.

Index	Models*		
	Single Factor	2-Factor Orthogonal	**2-Factor Oblique**
**GRTQ-Engagement**			
Comparative Fit Index (CFI)	0.876	0.551	**0.956**
Tucker-Lewis Index (TLI)	0.840	0.422	**0.942**
Standardized Root Mean Square Residual (SRMR)	0.081	0.306	**0.049**
**GRTQ-Perception**			
Comparative Fit Index (CFI)	0.833	0.824	**0.941**
Tucker-Lewis Index (TLI)	0.786	0.773	**0.935**
Standardized Root Mean Square Residual (SRMR)	0.157	0.210	**0.083**

**Diagonally weighted least squares (DWLS) was used to estimate the above model parameters. Full weight matrix was used to compute robust standard errors, and a mean-and variance-adjusted test statistics. The 2-factor oblique model (in BOLD) outperformed the other two models.*

Factor and construct validity (MI across gender and age for GRTQ-E and -P)

The percentage of respondents who had involved in and perceived as involving risk for each GRTQ items within each of the 4 subgroups: younger females, older females, younger males and older males was summarized in Supplementary Fig. S4. The results showed that all items within the 2 factors were perceived as RBs by more than 80% of the participants consistently across the 4 subgroups.

The MG-CFA results supported the measurement invariance of GRTQ-E and -P across these four groups of respondents. Even when the model was constrained to have the same items loaded on the same factors, with the same factor loadings and the likelihood of a shifting between response categories constrained to be equal across groups, the 2-factor oblique model still showed a reasonably good fit, as indicated by CFI = .959, TLI = .962 and SRMR = .07 for the engagement scale, and CFI = .959, TLI = .961 and SRMR = .085 for the GRTQ-P scale, as shown in Supplementary Table S6. These results suggested that structural and measurement invariance between different age and gender groups could be assumed, and the observed GRTQ scores across groups could be directly compared.

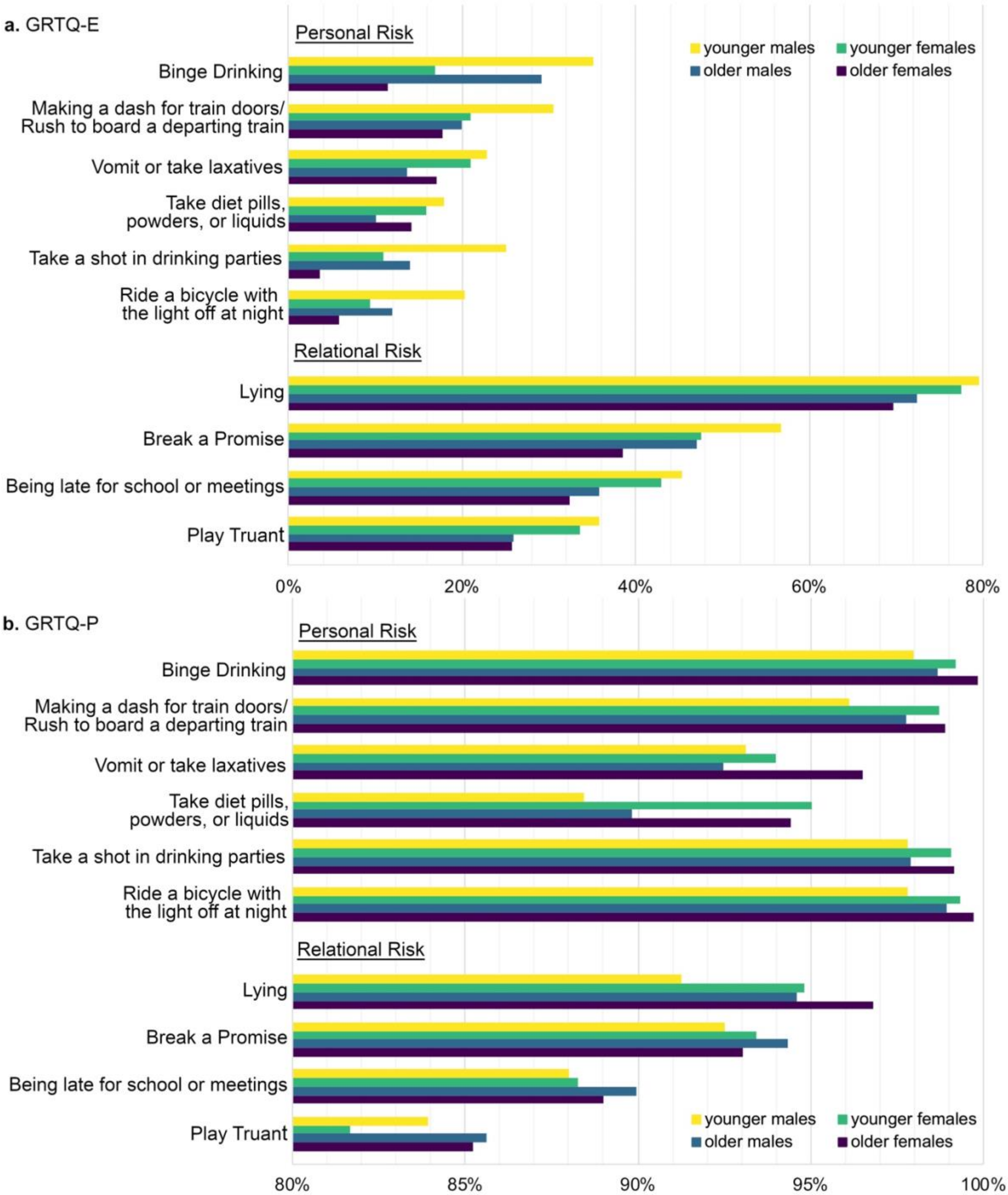

Supplementary Figure S4. Rating distributions for GRTQ-E and -P across four subgroups**.** (a.) The prevalence rates of the behaviours in GRTQ within each subgroup. The chart indicates the percentage of respondents who had given a response other than “Never” for GRTQ-E on each of the behaviours. (b.) The percentage of respondents who perceived the behaviours as involving risk, i.e., had chosen any options other than “Not at all risky” for GRTQ-P on each item within each subgroup.

Supplementary Table S6. Fit statistics of Multigroup confirmatory factor analyses (MG-CFA) using the 2-factor oblique model across four subsamples of younger females, older females, younger males, and older males.

Model fit index	TLI	CFI	ΔCFI	SRMR	ΔSRMR	$\Delta\chi^2$	df	*p*
**GRTQ-E**								
Configural model*	0.955	0.966		0.053			136	
Configural, threshold and loadings model**	0.962	0.959	-0.007	0.070	0.017	143.830	190	< .001
**GRTQ-P**								
Configural model*	0.936	0.952		0.084			136	
Configural, threshold and loadings model**	0.961	0.959	0.007	0.085	0.001	- 170.683	190	< .001

** Same items forced to load on the same factors across groups. ** Factor loadings and the likelihood of a shifting between response categories also constrained to be equal across groups.*

Concurrent validity

Bivariate correlations between the 11-item RIBS-U and the 10-item GRTQ-E are presented in Supplementary Table S7. Strong correlations were observed between the scorings in the subscales of the RIBS-U and corresponding subscales of the GRTQ-E, providing evidence of concurrent validity: Personal Risk ($r = .71$, $p < .001$), Social/Relational Risk ($r = .95$, $p < .001$).

Supplementary Table S7. Correlations between mean scores of the GRTQ-E and RIBS-U.

	**GRTQ-Engagement**		
	Full	Personal Risk	Relational Risk
**RIBS-U**[a]			
Full	.91***	.80***	.81***
Personal Risk	.65***	.71***	.41***
Social Risk	.90***	.67***	.95***

***** Pearson’s correlation coefficients, all p<.001.*

[a] *One RIBS-U original item, “Over-speed cycling/motorcycling”, in the Personal Risk subscale was not administered to participants and hence was not counted.*

Test-retest reliability

ICC values for both GRTQ-E, -P and all of their subscales were above .50, indicating a moderate to good reliability of GRTQ (Supplementary Table S8). Pearson's r values were above .60 for the GRTQ-E and its Personal Risk and Relational Risk subscales, and above .50 for the GRTQ-P and its subscales.

Supplementary Table S8. One-month test-retest reliability for the GRTQ-E, -P and all sub-scales (n = 99).

			**95% CI**	
**Scale**	**Pearson *r***	**ICC**	**Lower Bound**	**Upper Bound**
**GRTQ-Engagement**				
Full	.668**	.649	.520	.750
Personal Risk	.703**	.688	.569	.779
Relational Risk	.630**	.624	.488	.731
**GRTQ-Perception**				
Full	.584**	.584	.437	.700
Personal Risk	.542**	.543	.387	.668
Relational Risk	.587**	.588	.442	.704

*** p < 0.01 (2-tailed). ICCs were calculated using a Single-Rating, Absolute Agreement, 2-Way Mixed-Effects Model.*

Supplementary References

1. Korkmaz, S., Goksuluk, D. & Zararsiz, G. *MVN: An R Package for Assessing Multivariate Normality*. http://www.biosoft.hacettepe.edu.tr/MVN/.
2. Costello, A. B. & Osborne, J. Best practices in exploratory factor analysis: four recommendations for getting the most from your analysis. *Research, and Evaluation Practical Assessment, Research, and Evaluation* **10**, 7 (2005).
3. Baglin, J. Improving Your Exploratory Factor Analysis for Ordinal Data: A Demonstration Using FACTOR. *Practical Assessment, Research, and Evaluation* **19**, 5 (2014).
4. Bernstein, I. H. & Teng, G. Factoring Items and Factoring Scales Are Different: Spurious Evidence for Multidimensionality Due to Item Categorization. *Psychological Bulletin* **105**, 467–477 (1989).
5. Holgado-Tello, F. *et al.* Polychoric versus Pearson correlations in Exploratory and Confirmatory Factor Analysis with ordinal variables Effectiveness of training programs: a meta-analysis View project psicometria View project Polychoric versus Pearson correlations in exploratory and confirmatory factor analysis of ordinal variables. *Qual Quant* **44**, 153–166 (2010).
6. Morata-Ramírez, M. de los Á. & Holgado-Tello, F. P. Construct Validity of Likert Scales through Confirmatory Factor Analysis: A Simulation Study Comparing Different Methods of Estimation Based on Pearson and Polychoric Correlations. *International Journal of Social Science Studies* **1**, 54–61 (2013).
7. Olsson, U. Measuring Correlation in Ordered Two-Way Contingency Tables. *Journal of Marketing Research* **17**, 391–394 (1980).
8. Quiroga, A. Studies of the polychoric correlation and other correlation measures for ordinal variables. (University of Uppsala, 1994).
9. Costello, A. B. & Osborne, J. Best practices in exploratory factor analysis: four recommendations for getting the most from your analysis. *Research, and Evaluation Practical Assessment, Research, and Evaluation* **10**, 7 (2005).
10. Samuels, P. *Advice on Exploratory Factor Analysis*. http://www.open-access.bcu.ac.uk/id/eprint/6076 (2017).
11. Barbara G. Tabachnick & Linda S. Fidell. *Using Multivariate Statistics*. (Pearson, 2013).
12. Gadermann, A. M., Guhn, M. & Zumbo, B. D. Estimating ordinal reliability for Likert-type and ordinal item Estimating ordinal reliability for Likert-type and ordinal item response data: A conceptual, empirical, and practical guide response data: A conceptual, empirical, and practical guide. *Practical Assessment, Research, and Evaluation* **17**, 3 (2012).

13. Taber, K. S. The Use of Cronbach's Alpha When Developing and Reporting Research Instruments in Science Education. *Research in Science Education* **48**, 1273–1296 (2018).
14. Distefano, C. & Morgan, G. B. Structural Equation Modeling: A Multidisciplinary Journal A Comparison of Diagonal Weighted Least Squares Robust Estimation Techniques for Ordinal Data. *Taylor & Francis* **21**, 425–438 (2014).
15. Forero, C. G., Maydeu-Olivares, A. & Gallardo-Pujol, D. Factor Analysis with Ordinal Indicators: A Monte Carlo Study Comparing DWLS and ULS Estimation. *Structural Equation Modeling: A Multidisciplinary Journal* **16**, 625–641 (2009).
16. Muthén, B., du Toit, S. H. C. & Spisic, D. Robust inference using weighted least squares and quadratic estimating equations in latent variable modeling with categorical and continuous outcomes. *Unpublished technical report* (1997).
17. Savalei, V. & Rhemtulla, M. The performance of robust test statistics with categorical data. *Wiley Online Library* **66**, 201–223 (2013).
18. Thompson, B. *Exploratory and confirmatory factor analysis*. (2004).
19. Rosseel, Y. Lavaan: An R package for structural equation modeling. *Journal of Statistical Software* **48**, (2012).
20. Hu, L. T. & Bentler, P. M. Cutoff criteria for fit indexes in covariance structure analysis: Conventional criteria versus new alternatives. *Structural Equation Modeling: A Multidisciplinary Journal* **6**, 1–55 (1999).
21. Kline, R. B. Promise and pitfalls of structural equation modeling in gifted research. in *Methodologies for conducting research on giftedness*. 147–169 (American Psychological Association, 2010). doi:10.1037/12079-007.
22. Löwe, B. *et al.* A 4-item measure of depression and anxiety: Validation and standardization of the Patient Health Questionnaire-4 (PHQ-4) in the general population. *Journal of Affective Disorders* **122**, 86–95 (2010).
23. Tanaka, J. S. Multifaceted conceptions of fit in structural equation models. in *Testing Structural Equation Models* 10–39 (SAGE Publications, 1993).
24. Bentler, P. M. & Bonett, D. G. Significance tests and goodness of fit in the analysis of covariance structures. *Psychological Bulletin* **88**, 588–606 (1980).
25. Bentler, P. M. Comparative fit indexes in structural models. *Psychological Bulletin* **107**, 238–246 (1990).
26. Tucker, L. R. & Lewis, C. A reliability coefficient for maximum likelihood factor analysis. *Psychometrika* **38**, 1–10 (1973).
27. Kline, R. B. *Principles and Practice of Structural Equation Modeling.* . (The Guilford Press, 1998).
28. McDonald, R. P. & Ho, M. H. R. Principles and practice in reporting structural equation analyses. *Psychological Methods* **7**, 64–82 (2002).

29. Schumacker, R. & Lomax, R. *A beginner's guide to structural equation modeling*. (Psychology Press, 2004).
30. Maydeu-Olivares, A., Shi, D. & Rosseel, Y. Assessing Fit in Structural Equation Models: A Monte-Carlo Evaluation of RMSEA Versus SRMR Confidence Intervals and Tests of Close Fit. *Structural Equation Modeling: A Multidisciplinary Journal* **25**, 389–402 (2018).
31. Shi, D., Maydeu-Olivares, A. & Rosseel, Y. Assessing Fit in Ordinal Factor Analysis Models: SRMR vs. RMSEA. *Structural Equation Modeling: A Multidisciplinary Journal* **27**, 1–15 (2020).
32. Steiger, J. H. Structural Model Evaluation and Modification: An Interval Estimation Approach. *Multivariate Behavioral Research* **25**, 173–180 (1990).
33. Iacobucci, D. Everything you always wanted to know about SEM (structural equations modeling) but were afraid to ask. *Wiley Online Library* **19**, 673–680 (2009).
34. Pendergast, L. L., von der Embse, N., Kilgus, S. P. & Eklund, K. R. Measurement equivalence: A non-technical primer on categorical multi-group confirmatory factor analysis in school psychology. *Journal of School Psychology* **60**, 65–82 (2017).
35. Beauducel, A. & Herzberg, P. Y. On the performance of maximum likelihood versus means and variance adjusted weighted least squares estimation in CFA. *Structural Equation Modeling: A Multidisciplinary Journal* **13**, 186–203 (2006).
36. Chen, F. F. Sensitivity of goodness of fit indexes to lack of measurement invariance. *Structural Equation Modeling: A Multidisciplinary Journal* **14**, 464–504 (2007).
37. Chen, P.-Y., Wu, W., Watts, A., Forbush, K. & Johnson, P. *Strategies to deal with ordinal missing data for measurement invariance testing and specification searches-A comparison of commonly used methods*. https://kuscholarworks.ku.edu/handle/1808/27992 (2018).
38. Drasgow, F. & Kanfer, R. Equivalence of Psychological Measurement in Heterogeneous Populations Work Science Center View project. *Article in Journal of Applied Psychology* (1985) doi:10.1037/0021-9010.70.4.662.
39. Koh, K. H., Zumbo, B. D. & And Zumbo, B. D. Multi-Group Confirmatory Factor Analysis for Testing Measurement Invariance in Mixed Item Format Data. *Journal of Modern Applied Statistical Methods* **7**, 12 (2008).
40. Marsh, H. W., Balla, J. R. & McDonald, R. P. Goodness-of-Fit Indexes in Confirmatory Factor Analysis: The Effect of Sample Size. *Psychological Bulletin* **103**, 391–410 (1988).
41. Boateng, G. O., Neilands, T. B., Frongillo, E. A., Melgar-Quiñonez, H. R. & Young, S. L. Best Practices for Developing and Validating Scales for Health, Social, and Behavioral Research: A Primer. *Frontiers in Public Health* **6**, 149 (2018).

42. Laborde, S., Allen, M. S. & Guillén, F. Construct and concurrent validity of the short- and long-form versions of the trait emotional intelligence questionnaire. *Personality and Individual Differences* **101**, 232–235 (2016).
43. Koo, T. K. & Li, M. Y. A Guideline of Selecting and Reporting Intraclass Correlation Coefficients for Reliability Research. *Journal of Chiropractic Medicine* **15**, 155–163 (2016).
44. Mcgraw, K. O. Forming Inferences About Some Intraclass Correlation Coefficients Intrinsic motivation View project. *psycnet.apa.org* **1**, 30–46 (1996).
45. Fleiss JL. Reliability of Measurement. in *The Design and Analysis of Clinical Experiments* 1–32 (John Wiley & Sons, Ltd, 2011). doi:10.1002/9781118032923.ch1.
46. Wang, X. T., Zheng, R., Xuan, Y. H., Chen, J. & Li, S. Not all risks are created equal: A twin study and meta-analyses of risk taking across seven domains. *J Exp Psychol Gen* **145**, 1548–1560 (2016).
